# 2-[1-(4-*tert*-Butyl­phen­yl)-4,5-diphenyl-1*H*-imid­azol-2-yl]-4,6-di­chloro­phenol

**DOI:** 10.1107/S2414314620008706

**Published:** 2020-07-21

**Authors:** K. N. Shraddha, Noor Shahina Begum

**Affiliations:** a Department of Chemistry, Bangalore University, Jnana Bharathi Campus, Bangalore-560 056, Karnataka, India; University of Aberdeen, Scotland

**Keywords:** crystal structure, imidazole derivative, hydrogen bonding

## Abstract

In the title compound, C_31_H_26_Cl_2_N_2_O, the 4,6-di­chloro­phenol and the imidazole rings are almost coplanar, with a dihedral angle of 8.89 (6)° between them and an intra­molecular O—H⋯N hydrogen bond occurs between the rings. The dihedral angles subtended by the *tert*-butyl­phenyl ring and the two phenyl rings with the imidazole ring are 85.18 (9), 81.22 (9) and 19.00 (8)°, respectively. The methyl groups of the *tert*-butyl grouping are disordered over two sets of sites in 0.589 (5):0.411 (5) ratio. In the crystal, inversion dimers linked by pairs of weak C—H⋯Cl inter­actions generate *R*_2_^2^(24) loops.

## Abstract 2

An intra­molecular O—H⋯N hydrogen bond helps to ensure near coplanarity of the 4,6-di­chloro­phenol and imidazole rings in the title compound.

## Graphical abstract

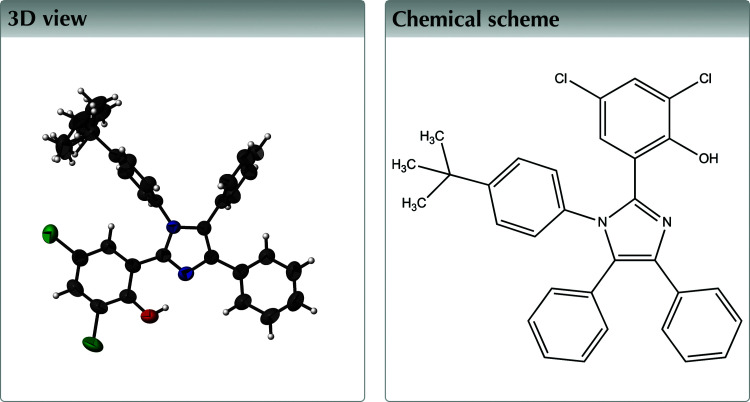

## Structure description

**Figure 1:**
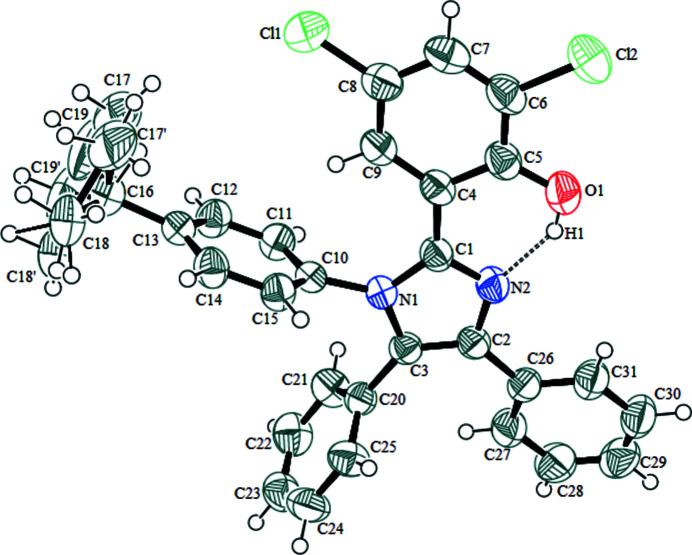
The mol­ecular structure of the title compound with displacement ellipsoids drawn at the 50% probability level. The hydrogen bond is indicated by a double-dashed line.

Imidazole is an active part of anti­fungal compounds like clotrimazole, ketoconazole, miconazole, isoconazole and econazole (Bastide *et al.*, 1982 ▸). As part of our studies of imidazole derivatives, we now report the synthesis and crystal structure of the title compound, which consists of an imidazole ring bearing C10–C15 *tert*-butyl­phenyl, C4–C9 4,6-di­chloro­phenol and C20–C25 and C26–C31 phenyl rings (Fig. 1 ▸). The imidazole and the 4,6-di­chloro­phenol rings are close to coplanar with a dihedral angle of 8.89 (6)°. The dihedral angles between the imidazole ring and the *tert*-butyl­phenyl ring and the C20- and C26-phenyl rings are 85.18 (9), 81.22 (9) and 19.00 (8)°, respectively. The C17, C18 and C19 carbon atoms of the *tert*-butyl group are disordered over two sets of sites in a 0.59:0.41 ratio. A short and presumably strong intra­molecular O1—H1⋯N2 hydrogen bond is formed between the O1 atom of the 4,6-di­chloro­phenol ring and atom N2 of the imidazole ring (Table 1 ▸, Fig. 1 ▸), forming an *S*(6) ring, which helps to establish the near coplanarity of the imidazole and phenol rings.

**Figure 2:**
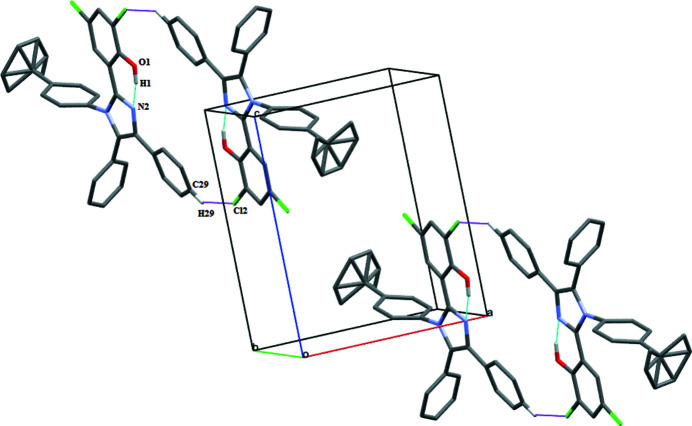
Unit-cell packing of the title compound showing intra­molecular O—H⋯N and inter­molecular C—H⋯Cl inter­actions with dotted lines. H atoms not involved in hydrogen bonding have been excluded.

In the crystal, pairwise C—H⋯Cl inter­actions involving atoms C29 of the phenyl ring and the Cl2 atom of the phenol group generate centrosymmetric 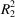 (24) loops (Table 1 ▸, Fig. 2 ▸).

## Synthesis and crystallization

The title compound was synthesized by the one-pot reaction of benzil (10 mmol), 4-*tert*-butyl­aniline (10 mmol) and 3,5-di­chloro-2-hy­droxy­benzaldehyde (10 mmol) with ammonium acetate (10 mmol) in a glacial acetic acid (20 ml) medium. The mixture was refluxed for 6 h at 343 K, the progress of the reaction being monitored by TLC. After completion of the reaction, the mixture was cooled to room temperature and poured into 100 ml of ice-cold water. The resulting precipitate was filtered, dried and further purified by column chromatography (7:3 petroleum ether:ethyl acetate) and isolated in good yield (85%). Colourless needles were recrystallized from methanol solution.

## Refinement

Crystal data, data collection and structure refinement details are summarized in Table 2 ▸.

## Supplementary Material

Crystal structure: contains datablock(s) global, I. DOI: 10.1107/S2414314620008706/hb4352sup1.cif

Structure factors: contains datablock(s) I. DOI: 10.1107/S2414314620008706/hb4352Isup2.hkl

Supporting information file. DOI: 10.1107/S2414314620008706/hb4352Isup3.cml

CCDC reference: 2012170

Additional supporting information:  crystallographic information; 3D view; checkCIF report

## Figures and Tables

**Table 1:** Hydrogen-bond geometry (Å, °)

*D*—H⋯*A*	*D*—H	H⋯*A*	*D*⋯*A*	*D*—H⋯*A*
O1—H1⋯N2	0.82	1.81	2.541 (2)	147
C29—H29⋯Cl2^i^	0.93	2.97	3.705 (3)	137

Symmetry code: (i) 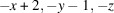 .

**Table 2:** Experimental details

Crystal data	
Chemical formula	C_31_H_26_Cl_2_N_2_O
*M*_r_	513.44
Crystal system, space group	Triclinic, *P* 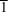
Temperature (K)	446
*a*, *b*, *c* (Å)	9.8084 (10), 11.6806 (11), 12.0754 (13)
α, β, γ (°)	77.474 (6), 85.686 (6), 80.742 (6)
*V* (Å^3^)	1331.8 (2)
*Z*	2
Radiation type	Mo *K*α
μ (mm^−1^)	0.27
Crystal size (mm)	0.15 × 0.13 × 0.12

Data collection	
Diffractometer	Bruker SMART APEX CCD
Absorption correction	Multi-scan (*SADABS*; Bruker, 1998 ▸)
*T*_min_, *T*_max_	0.862, 0.960
No. of measured, independent and observed [*I* > 2σ(*I*)] reflections	19148, 4634, 3610
*R*_int_	0.037
(sin θ/λ)_max_ (Å^−1^)	0.595

Refinement	
*R*[*F* ^2^ > 2σ(*F* ^2^)], *wR*(*F* ^2^), *S*	0.063, 0.221, 1.14
No. of reflections	4634
No. of parameters	343
H-atom treatment	H-atom parameters constrained
Δρ_max_, Δρ_min_ (e Å^−3^)	0.45, −0.51

Computer programs: *SMART* and *SAINT-Plus* (Bruker, 1998 ▸), *SHELXS97* (Sheldrick, 2008 ▸), *SHELXL2018* (Sheldrick, 2015 ▸) and *ORTEP-3 for Windows* (Farrugia, 2012 ▸).

## full crystallographic data

### Crystal data

**Table:**

C_31_H_26_Cl_2_N_2_O	*Z* = 2
*M**_r_* = 513.44	*F*(000) = 536
Triclinic, *P*1	*D*_x_ = 1.280 Mg m^−^^3^
*a* = 9.8084 (10) Å	Mo *K*α radiation, λ = 0.71073 Å
*b* = 11.6806 (11) Å	Cell parameters from 4634 reflections
*c* = 12.0754 (13) Å	θ = 3.3–30.8°
α = 77.474 (6)°	µ = 0.27 mm^−^^1^
β = 85.686 (6)°	*T* = 446 K
γ = 80.742 (6)°	Needle, colorless
*V* = 1331.8 (2) Å^3^	0.15 × 0.13 × 0.12 mm

### Data collection

**Table:**

Bruker SMART APEX CCD diffractometer	3610 reflections with *I* > 2σ(*I*)
Radiation source: fine-focus sealed tube	*R*_int_ = 0.037
ω scans	θ_max_ = 25.0°, θ_min_ = 3.3°
Absorption correction: multi-scan (SADABS; Bruker, 1998)	*h* = −11→11
*T*_min_ = 0.862, *T*_max_ = 0.960	*k* = −13→13
19148 measured reflections	*l* = −14→14
4634 independent reflections	

### Refinement

**Table:**

Refinement on *F*^2^	Primary atom site location: structure-invariant direct methods
Least-squares matrix: full	Secondary atom site location: difference Fourier map
*R*[*F*^2^ > 2σ(*F*^2^)] = 0.063	Hydrogen site location: inferred from neighbouring sites
*wR*(*F*^2^) = 0.221	H-atom parameters constrained
*S* = 1.14	*w* = 1/[σ^2^(*F*_o_^2^) + (0.1532*P*)^2^ + 0.0304*P*] where *P* = (*F*_o_^2^ + 2*F*_c_^2^)/3
4634 reflections	(Δ/σ)_max_ < 0.001
343 parameters	Δρ_max_ = 0.45 e Å^−^^3^
0 restraints	Δρ_min_ = −0.51 e Å^−^^3^

### Special details

**Table:**

Geometry. All esds (except the esd in the dihedral angle between two l.s. planes) are estimated using the full covariance matrix. The cell esds are taken into account individually in the estimation of esds in distances, angles and torsion angles; correlations between esds in cell parameters are only used when they are defined by crystal symmetry. An approximate (isotropic) treatment of cell esds is used for estimating esds involving l.s. planes.

### Fractional atomic coordinates and isotropic or equivalent isotropic displacement parameters (Å^2^)

**Table:**

	*x*	*y*	*z*	*U*_iso_*/*U*_eq_	Occ. (<1)
Cl1	0.68151 (10)	0.02403 (8)	0.47335 (7)	0.0842 (3)	
Cl2	0.83657 (11)	−0.42569 (7)	0.42892 (8)	0.0941 (4)	
N2	0.84180 (19)	−0.16232 (18)	0.01276 (18)	0.0513 (5)	
N1	0.74765 (18)	0.02248 (16)	0.00830 (16)	0.0443 (5)	
C10	0.6835 (2)	0.12908 (19)	0.04413 (18)	0.0420 (5)	
O1	0.8521 (2)	−0.32598 (16)	0.19026 (18)	0.0683 (5)	
H1	0.849884	−0.296243	0.122230	0.102*	
C1	0.7867 (2)	−0.0865 (2)	0.0763 (2)	0.0475 (5)	
C4	0.7737 (2)	−0.1209 (2)	0.2000 (2)	0.0493 (6)	
C9	0.7350 (3)	−0.0408 (2)	0.2719 (2)	0.0565 (6)	
H9	0.714640	0.039674	0.240357	0.068*	
C20	0.7522 (2)	0.1150 (2)	−0.19884 (19)	0.0457 (5)	
C3	0.7825 (2)	0.0130 (2)	−0.10358 (19)	0.0446 (5)	
C13	0.5662 (2)	0.3168 (2)	0.1463 (2)	0.0487 (6)	
C2	0.8415 (2)	−0.1021 (2)	−0.0989 (2)	0.0472 (5)	
C11	0.5434 (2)	0.1483 (2)	0.0672 (2)	0.0494 (6)	
H11	0.487336	0.098558	0.048844	0.059*	
C6	0.7951 (3)	−0.2766 (2)	0.3677 (2)	0.0604 (7)	
C12	0.4858 (2)	0.2410 (2)	0.1173 (2)	0.0535 (6)	
H12	0.390733	0.253123	0.132195	0.064*	
C26	0.9009 (2)	−0.1638 (2)	−0.1902 (2)	0.0521 (6)	
C5	0.8068 (2)	−0.2424 (2)	0.2500 (2)	0.0537 (6)	
C15	0.7649 (2)	0.2069 (2)	0.0664 (2)	0.0518 (6)	
H15	0.859112	0.197337	0.047397	0.062*	
C7	0.7564 (3)	−0.1977 (3)	0.4363 (2)	0.0637 (7)	
H7	0.750008	−0.223624	0.514664	0.076*	
C14	0.7055 (3)	0.2987 (2)	0.1168 (2)	0.0559 (6)	
H14	0.761210	0.350459	0.131681	0.067*	
C8	0.7265 (3)	−0.0782 (3)	0.3877 (2)	0.0608 (7)	
C23	0.7007 (3)	0.3025 (3)	−0.3821 (2)	0.0688 (8)	
H23	0.683701	0.364908	−0.444302	0.083*	
C16	0.5035 (3)	0.4127 (3)	0.2117 (3)	0.0635 (7)	
C27	0.9426 (3)	−0.1033 (3)	−0.2964 (2)	0.0655 (7)	
H27	0.930101	−0.020850	−0.312384	0.079*	
C31	0.9224 (3)	−0.2866 (2)	−0.1699 (3)	0.0616 (7)	
H31	0.895750	−0.329589	−0.099589	0.074*	
C25	0.6370 (3)	0.1268 (3)	−0.2620 (2)	0.0617 (7)	
H25	0.576498	0.071204	−0.243154	0.074*	
C24	0.6116 (3)	0.2202 (3)	−0.3524 (3)	0.0708 (8)	
H24	0.533302	0.227905	−0.393903	0.085*	
C21	0.8394 (3)	0.1993 (3)	−0.2284 (3)	0.0628 (7)	
H21	0.916475	0.193590	−0.185982	0.075*	
C22	0.8134 (3)	0.2921 (3)	−0.3203 (3)	0.0713 (8)	
H22	0.873597	0.347898	−0.339950	0.086*	
C28	1.0019 (3)	−0.1632 (3)	−0.3776 (3)	0.0777 (9)	
H28	1.029066	−0.121011	−0.448159	0.093*	
C29	1.0216 (3)	−0.2846 (3)	−0.3563 (3)	0.0772 (9)	
H29	1.061113	−0.324753	−0.412158	0.093*	
C30	0.9828 (3)	−0.3463 (3)	−0.2524 (3)	0.0734 (8)	
H30	0.997088	−0.428761	−0.237014	0.088*	
C18	0.3485 (11)	0.4343 (10)	0.2197 (11)	0.0954 (19)	0.411 (5)
H18A	0.314639	0.361124	0.252109	0.143*	0.411 (5)
H18B	0.313739	0.464925	0.145138	0.143*	0.411 (5)
H18C	0.318046	0.490703	0.266842	0.143*	0.411 (5)
C18'	0.3761 (8)	0.4882 (7)	0.1474 (7)	0.0954 (19)	0.589 (5)
H18D	0.310146	0.437512	0.141677	0.143*	0.589 (5)
H18E	0.405720	0.525813	0.072679	0.143*	0.589 (5)
H18F	0.334166	0.547566	0.188362	0.143*	0.589 (5)
C17	0.5525 (19)	0.3722 (13)	0.3351 (10)	0.128 (3)	0.411 (5)
H17A	0.491571	0.404759	0.390135	0.191*	0.411 (5)
H17B	0.628819	0.319934	0.372610	0.191*	0.411 (5)
H17C	0.503172	0.328690	0.296726	0.191*	0.411 (5)
C17'	0.4401 (12)	0.3491 (9)	0.3245 (7)	0.128 (3)	0.589 (5)
H17D	0.394795	0.286780	0.310901	0.191*	0.589 (5)
H17E	0.374022	0.404937	0.355793	0.191*	0.589 (5)
H17F	0.511909	0.316061	0.377054	0.191*	0.589 (5)
C19	0.6053 (15)	0.4698 (11)	0.2511 (13)	0.113 (3)	0.411 (5)
H19A	0.564836	0.507319	0.311546	0.169*	0.411 (5)
H19B	0.635841	0.528419	0.189603	0.169*	0.411 (5)
H19C	0.682744	0.411619	0.278258	0.169*	0.411 (5)
C19'	0.5770 (10)	0.5256 (7)	0.1748 (9)	0.113 (3)	0.589 (5)
H19D	0.533195	0.585609	0.214414	0.169*	0.589 (5)
H19E	0.570027	0.555101	0.094509	0.169*	0.589 (5)
H19F	0.672616	0.505214	0.193012	0.169*	0.589 (5)

### Atomic displacement parameters (Å^2^)

**Table:**

	*U*^11^	*U*^22^	*U*^33^	*U*^12^	*U*^13^	*U*^23^
Cl1	0.1116 (7)	0.0822 (6)	0.0570 (5)	−0.0001 (5)	−0.0033 (4)	−0.0208 (4)
Cl2	0.1401 (8)	0.0527 (5)	0.0788 (6)	−0.0159 (5)	−0.0115 (5)	0.0128 (4)
N2	0.0478 (10)	0.0470 (11)	0.0576 (12)	0.0012 (8)	−0.0050 (8)	−0.0125 (9)
N1	0.0457 (9)	0.0400 (10)	0.0458 (11)	−0.0017 (7)	−0.0047 (8)	−0.0081 (8)
C10	0.0447 (10)	0.0393 (11)	0.0406 (12)	−0.0025 (8)	−0.0061 (8)	−0.0062 (9)
O1	0.0902 (13)	0.0435 (10)	0.0673 (12)	−0.0044 (9)	−0.0039 (10)	−0.0069 (9)
C1	0.0452 (11)	0.0445 (12)	0.0514 (14)	−0.0033 (9)	−0.0064 (10)	−0.0082 (10)
C4	0.0449 (11)	0.0477 (13)	0.0525 (14)	−0.0035 (9)	−0.0074 (10)	−0.0050 (11)
C9	0.0626 (14)	0.0512 (14)	0.0508 (14)	0.0002 (11)	−0.0070 (11)	−0.0049 (11)
C20	0.0438 (11)	0.0482 (13)	0.0440 (12)	−0.0029 (9)	0.0008 (9)	−0.0111 (10)
C3	0.0405 (10)	0.0480 (13)	0.0458 (12)	−0.0053 (9)	−0.0034 (9)	−0.0108 (10)
C13	0.0543 (12)	0.0444 (12)	0.0454 (13)	−0.0025 (10)	−0.0056 (10)	−0.0073 (10)
C2	0.0422 (11)	0.0497 (13)	0.0493 (13)	−0.0021 (9)	−0.0060 (9)	−0.0116 (10)
C11	0.0441 (11)	0.0456 (13)	0.0607 (15)	−0.0089 (9)	−0.0047 (10)	−0.0131 (11)
C6	0.0645 (14)	0.0499 (14)	0.0615 (16)	−0.0121 (11)	−0.0081 (12)	0.0040 (12)
C12	0.0410 (11)	0.0513 (13)	0.0674 (16)	−0.0028 (10)	−0.0007 (10)	−0.0138 (12)
C26	0.0402 (11)	0.0576 (14)	0.0618 (16)	0.0004 (10)	−0.0085 (10)	−0.0231 (12)
C5	0.0527 (12)	0.0462 (13)	0.0610 (16)	−0.0105 (10)	−0.0077 (11)	−0.0043 (11)
C15	0.0405 (11)	0.0563 (14)	0.0608 (15)	−0.0080 (10)	−0.0011 (10)	−0.0164 (12)
C7	0.0655 (15)	0.0681 (18)	0.0524 (15)	−0.0153 (13)	−0.0084 (12)	0.0045 (13)
C14	0.0533 (13)	0.0519 (14)	0.0674 (16)	−0.0138 (10)	−0.0048 (11)	−0.0182 (12)
C8	0.0591 (14)	0.0653 (16)	0.0551 (15)	−0.0051 (12)	−0.0081 (11)	−0.0076 (13)
C23	0.0851 (19)	0.0612 (17)	0.0505 (15)	0.0017 (14)	0.0043 (14)	−0.0023 (13)
C16	0.0713 (16)	0.0565 (15)	0.0638 (17)	−0.0011 (12)	−0.0022 (13)	−0.0215 (13)
C27	0.0728 (16)	0.0600 (16)	0.0613 (17)	0.0010 (13)	0.0013 (13)	−0.0165 (13)
C31	0.0608 (14)	0.0574 (15)	0.0695 (17)	−0.0049 (12)	−0.0015 (12)	−0.0227 (13)
C25	0.0581 (14)	0.0676 (17)	0.0586 (16)	−0.0150 (12)	−0.0105 (12)	−0.0049 (13)
C24	0.0754 (17)	0.0769 (19)	0.0556 (16)	−0.0049 (15)	−0.0203 (13)	−0.0031 (14)
C21	0.0549 (13)	0.0623 (16)	0.0690 (18)	−0.0136 (12)	−0.0048 (12)	−0.0048 (13)
C22	0.0755 (18)	0.0624 (18)	0.0722 (19)	−0.0179 (14)	0.0091 (15)	−0.0042 (15)
C28	0.0844 (19)	0.088 (2)	0.0586 (18)	−0.0001 (17)	0.0062 (15)	−0.0238 (16)
C29	0.0728 (17)	0.089 (2)	0.074 (2)	0.0046 (16)	0.0006 (15)	−0.0403 (18)
C30	0.0730 (17)	0.0613 (17)	0.091 (2)	0.0015 (14)	−0.0071 (15)	−0.0337 (17)
C18	0.104 (4)	0.077 (4)	0.101 (5)	0.029 (3)	−0.009 (4)	−0.040 (3)
C18'	0.104 (4)	0.077 (4)	0.101 (5)	0.029 (3)	−0.009 (4)	−0.040 (3)
C17	0.195 (9)	0.114 (5)	0.058 (3)	0.024 (7)	0.023 (6)	−0.029 (3)
C17'	0.195 (9)	0.114 (5)	0.058 (3)	0.024 (7)	0.023 (6)	−0.029 (3)
C19	0.154 (6)	0.066 (4)	0.132 (7)	−0.031 (5)	0.038 (6)	−0.058 (4)
C19'	0.154 (6)	0.066 (4)	0.132 (7)	−0.031 (5)	0.038 (6)	−0.058 (4)

### Geometric parameters (Å, º)

**Table:**

Cl1—C8	1.729 (3)	C16—C18	1.500 (11)
Cl2—C6	1.733 (3)	C16—C17'	1.542 (9)
N2—C1	1.321 (3)	C16—C17	1.550 (14)
N2—C2	1.379 (3)	C16—C18'	1.561 (8)
N1—C1	1.370 (3)	C16—C19'	1.567 (8)
N1—C3	1.392 (3)	C27—C28	1.368 (4)
N1—C10	1.440 (3)	C27—H27	0.9300
C10—C11	1.374 (3)	C31—C30	1.381 (4)
C10—C15	1.382 (3)	C31—H31	0.9300
O1—C5	1.338 (3)	C25—C24	1.371 (4)
O1—H1	0.8200	C25—H25	0.9300
C1—C4	1.461 (3)	C24—H24	0.9300
C4—C9	1.400 (4)	C21—C22	1.380 (4)
C4—C5	1.412 (3)	C21—H21	0.9300
C9—C8	1.372 (4)	C22—H22	0.9300
C9—H9	0.9300	C28—C29	1.370 (5)
C20—C21	1.380 (3)	C28—H28	0.9300
C20—C25	1.383 (4)	C29—C30	1.365 (5)
C20—C3	1.474 (3)	C29—H29	0.9300
C3—C2	1.367 (3)	C30—H30	0.9300
C13—C14	1.380 (3)	C18—H18A	0.9600
C13—C12	1.389 (3)	C18—H18B	0.9600
C13—C16	1.532 (3)	C18—H18C	0.9600
C2—C26	1.479 (3)	C18'—H18D	0.9600
C11—C12	1.376 (3)	C18'—H18E	0.9600
C11—H11	0.9300	C18'—H18F	0.9600
C6—C7	1.360 (4)	C17—C19	1.49 (2)
C6—C5	1.392 (4)	C17—H17A	0.9600
C12—H12	0.9300	C17—H17B	0.9600
C26—C31	1.386 (4)	C17—H17C	0.9600
C26—C27	1.390 (4)	C17'—H17D	0.9600
C15—C14	1.374 (4)	C17'—H17E	0.9600
C15—H15	0.9300	C17'—H17F	0.9600
C7—C8	1.384 (4)	C19—H19A	0.9600
C7—H7	0.9300	C19—H19B	0.9600
C14—H14	0.9300	C19—H19C	0.9600
C23—C22	1.355 (5)	C19'—H19D	0.9600
C23—C24	1.376 (4)	C19'—H19E	0.9600
C23—H23	0.9300	C19'—H19F	0.9600
C16—C19	1.445 (14)		
			
C1—N2—C2	107.9 (2)	C28—C27—C26	121.0 (3)
C1—N1—C3	107.68 (19)	C28—C27—H27	119.5
C1—N1—C10	127.05 (19)	C26—C27—H27	119.5
C3—N1—C10	125.27 (18)	C30—C31—C26	121.1 (3)
C11—C10—C15	119.5 (2)	C30—C31—H31	119.5
C11—C10—N1	120.52 (19)	C26—C31—H31	119.5
C15—C10—N1	119.68 (18)	C24—C25—C20	120.2 (3)
C5—O1—H1	109.5	C24—C25—H25	119.9
N2—C1—N1	109.5 (2)	C20—C25—H25	119.9
N2—C1—C4	122.1 (2)	C25—C24—C23	120.6 (3)
N1—C1—C4	128.3 (2)	C25—C24—H24	119.7
C9—C4—C5	118.1 (2)	C23—C24—H24	119.7
C9—C4—C1	124.2 (2)	C20—C21—C22	120.6 (3)
C5—C4—C1	117.7 (2)	C20—C21—H21	119.7
C8—C9—C4	121.6 (3)	C22—C21—H21	119.7
C8—C9—H9	119.2	C23—C22—C21	120.4 (3)
C4—C9—H9	119.2	C23—C22—H22	119.8
C21—C20—C25	118.5 (2)	C21—C22—H22	119.8
C21—C20—C3	120.8 (2)	C27—C28—C29	120.7 (3)
C25—C20—C3	120.6 (2)	C27—C28—H28	119.7
C2—C3—N1	105.9 (2)	C29—C28—H28	119.7
C2—C3—C20	132.6 (2)	C30—C29—C28	119.5 (3)
N1—C3—C20	121.47 (19)	C30—C29—H29	120.2
C14—C13—C12	116.8 (2)	C28—C29—H29	120.2
C14—C13—C16	121.8 (2)	C29—C30—C31	120.2 (3)
C12—C13—C16	121.3 (2)	C29—C30—H30	119.9
C3—C2—N2	109.0 (2)	C31—C30—H30	119.9
C3—C2—C26	130.7 (2)	C16—C18—H18A	109.5
N2—C2—C26	120.2 (2)	C16—C18—H18B	109.5
C10—C11—C12	120.1 (2)	H18A—C18—H18B	109.5
C10—C11—H11	120.0	C16—C18—H18C	109.5
C12—C11—H11	120.0	H18A—C18—H18C	109.5
C7—C6—C5	122.7 (2)	H18B—C18—H18C	109.5
C7—C6—Cl2	119.0 (2)	C16—C18'—H18D	109.5
C5—C6—Cl2	118.3 (2)	C16—C18'—H18E	109.5
C11—C12—C13	121.6 (2)	H18D—C18'—H18E	109.5
C11—C12—H12	119.2	C16—C18'—H18F	109.5
C13—C12—H12	119.2	H18D—C18'—H18F	109.5
C31—C26—C27	117.5 (2)	H18E—C18'—H18F	109.5
C31—C26—C2	120.1 (2)	C19—C17—C16	56.8 (8)
C27—C26—C2	122.4 (2)	C19—C17—H17A	109.5
O1—C5—C6	118.3 (2)	C16—C17—H17A	114.2
O1—C5—C4	123.3 (2)	C19—C17—H17B	109.5
C6—C5—C4	118.4 (2)	C16—C17—H17B	136.3
C14—C15—C10	119.4 (2)	H17A—C17—H17B	109.5
C14—C15—H15	120.3	C19—C17—H17C	109.5
C10—C15—H15	120.3	C16—C17—H17C	54.2
C6—C7—C8	119.1 (3)	H17A—C17—H17C	109.5
C6—C7—H7	120.5	H17B—C17—H17C	109.5
C8—C7—H7	120.5	C16—C17'—H17D	109.5
C15—C14—C13	122.4 (2)	C16—C17'—H17E	109.5
C15—C14—H14	118.8	H17D—C17'—H17E	109.5
C13—C14—H14	118.8	C16—C17'—H17F	109.5
C9—C8—C7	120.1 (3)	H17D—C17'—H17F	109.5
C9—C8—Cl1	120.1 (2)	H17E—C17'—H17F	109.5
C7—C8—Cl1	119.9 (2)	C16—C19—C17	63.9 (8)
C22—C23—C24	119.6 (3)	C16—C19—H19A	109.5
C22—C23—H23	120.2	C17—C19—H19A	76.2
C24—C23—H23	120.2	C16—C19—H19B	109.5
C19—C16—C18	131.4 (6)	C17—C19—H19B	172.8
C19—C16—C13	113.7 (5)	H19A—C19—H19B	109.5
C18—C16—C13	114.8 (4)	C16—C19—H19C	109.5
C13—C16—C17'	107.0 (4)	C17—C19—H19C	71.7
C19—C16—C17	59.3 (9)	H19A—C19—H19C	109.5
C18—C16—C17	106.7 (8)	H19B—C19—H19C	109.5
C13—C16—C17	107.8 (5)	C16—C19'—H19D	109.5
C13—C16—C18'	108.4 (3)	C16—C19'—H19E	109.5
C17'—C16—C18'	103.9 (5)	H19D—C19'—H19E	109.5
C13—C16—C19'	111.1 (3)	C16—C19'—H19F	109.5
C17'—C16—C19'	134.6 (6)	H19D—C19'—H19F	109.5
C18'—C16—C19'	86.8 (6)	H19E—C19'—H19F	109.5
			
C1—N1—C10—C11	−82.3 (3)	C9—C4—C5—C6	2.0 (3)
C3—N1—C10—C11	98.5 (3)	C1—C4—C5—C6	179.9 (2)
C1—N1—C10—C15	91.3 (3)	C11—C10—C15—C14	3.5 (4)
C3—N1—C10—C15	−88.0 (3)	N1—C10—C15—C14	−170.1 (2)
C2—N2—C1—N1	1.1 (2)	C5—C6—C7—C8	0.3 (4)
C2—N2—C1—C4	−178.75 (19)	Cl2—C6—C7—C8	178.4 (2)
C3—N1—C1—N2	−0.7 (2)	C10—C15—C14—C13	−0.2 (4)
C10—N1—C1—N2	179.92 (19)	C12—C13—C14—C15	−3.2 (4)
C3—N1—C1—C4	179.1 (2)	C16—C13—C14—C15	174.6 (2)
C10—N1—C1—C4	−0.3 (3)	C4—C9—C8—C7	−0.1 (4)
N2—C1—C4—C9	170.3 (2)	C4—C9—C8—Cl1	178.79 (19)
N1—C1—C4—C9	−9.5 (4)	C6—C7—C8—C9	0.6 (4)
N2—C1—C4—C5	−7.5 (3)	C6—C7—C8—Cl1	−178.3 (2)
N1—C1—C4—C5	172.7 (2)	C14—C13—C16—C19	−7.5 (8)
C5—C4—C9—C8	−1.2 (4)	C12—C13—C16—C19	170.1 (7)
C1—C4—C9—C8	−179.0 (2)	C14—C13—C16—C18	170.2 (6)
C1—N1—C3—C2	0.1 (2)	C12—C13—C16—C18	−12.2 (7)
C10—N1—C3—C2	179.45 (18)	C14—C13—C16—C17'	−119.1 (5)
C1—N1—C3—C20	179.06 (18)	C12—C13—C16—C17'	58.6 (6)
C10—N1—C3—C20	−1.6 (3)	C14—C13—C16—C17	−71.2 (8)
C21—C20—C3—C2	−98.9 (3)	C12—C13—C16—C17	106.5 (7)
C25—C20—C3—C2	79.8 (3)	C14—C13—C16—C18'	129.4 (4)
C21—C20—C3—N1	82.4 (3)	C12—C13—C16—C18'	−52.9 (5)
C25—C20—C3—N1	−98.9 (3)	C14—C13—C16—C19'	35.7 (6)
N1—C3—C2—N2	0.6 (2)	C12—C13—C16—C19'	−146.6 (5)
C20—C3—C2—N2	−178.3 (2)	C31—C26—C27—C28	0.4 (4)
N1—C3—C2—C26	−178.7 (2)	C2—C26—C27—C28	177.6 (2)
C20—C3—C2—C26	2.5 (4)	C27—C26—C31—C30	−0.1 (4)
C1—N2—C2—C3	−1.0 (2)	C2—C26—C31—C30	−177.4 (2)
C1—N2—C2—C26	178.30 (19)	C21—C20—C25—C24	0.5 (4)
C15—C10—C11—C12	−3.3 (4)	C3—C20—C25—C24	−178.3 (3)
N1—C10—C11—C12	170.3 (2)	C20—C25—C24—C23	0.7 (5)
C10—C11—C12—C13	−0.2 (4)	C22—C23—C24—C25	−1.1 (5)
C14—C13—C12—C11	3.4 (4)	C25—C20—C21—C22	−1.2 (4)
C16—C13—C12—C11	−174.4 (2)	C3—C20—C21—C22	177.5 (2)
C3—C2—C26—C31	−163.2 (2)	C24—C23—C22—C21	0.4 (5)
N2—C2—C26—C31	17.6 (3)	C20—C21—C22—C23	0.8 (5)
C3—C2—C26—C27	19.6 (4)	C26—C27—C28—C29	0.0 (5)
N2—C2—C26—C27	−159.5 (2)	C27—C28—C29—C30	−0.6 (5)
C7—C6—C5—O1	176.8 (2)	C28—C29—C30—C31	0.9 (5)
Cl2—C6—C5—O1	−1.3 (3)	C26—C31—C30—C29	−0.5 (4)
C7—C6—C5—C4	−1.6 (4)	C18—C16—C17—C19	−128.7 (8)
Cl2—C6—C5—C4	−179.73 (18)	C13—C16—C17—C19	107.5 (6)
C9—C4—C5—O1	−176.3 (2)	C18—C16—C19—C17	85.5 (11)
C1—C4—C5—O1	1.6 (3)	C13—C16—C19—C17	−97.3 (7)

### Hydrogen-bond geometry (Å, º)

**Table:**

*D*—H···*A*	*D*—H	H···*A*	*D*···*A*	*D*—H···*A*
O1—H1···N2	0.82	1.81	2.541 (2)	147
C29—H29···Cl2^i^	0.93	2.97	3.705 (3)	137

Symmetry code: (i) −*x*+2, −*y*−1, −*z*.
